# Catabolism and interactions of uncultured organisms shaped by eco-thermodynamics in methanogenic bioprocesses

**DOI:** 10.1186/s40168-020-00885-y

**Published:** 2020-07-24

**Authors:** Masaru K. Nobu, Takashi Narihiro, Ran Mei, Yoichi Kamagata, Patrick K. H. Lee, Po-Heng Lee, Michael J. McInerney, Wen-Tso Liu

**Affiliations:** 1 Department of Civil and Environmental Engineering, University of Illinois at Urbana-Champaign, 205 N. Mathews Ave, Urbana, IL 61801 USA; 2 Bioproduction Research Institute, National Institute of Advanced Industrial Science and Technology, Tsukuba, Japan; 3 School of Energy and Environment, City University of Hong Kong, Kowloon, HK Hong Kong; 4 Department of Civil and Environmental Engineering, Imperial College, London, UK; 5 Department of Microbiology and Plant Biology, University of Oklahoma, Norman, Oklahoma USA

**Keywords:** Catabolism, Interactions, Uncultured organisms, Eco-thermodynamics, Methanogenic bioprocesses

## Abstract

**Background:**

Current understanding of the carbon cycle in methanogenic environments involves trophic interactions such as interspecies H_2_ transfer between organotrophs and methanogens. However, many metabolic processes are thermodynamically sensitive to H_2_ accumulation and can be inhibited by H_2_ produced from co-occurring metabolisms. Strategies for driving thermodynamically competing metabolisms in methanogenic environments remain unexplored.

**Results:**

To uncover how anaerobes combat this H_2_ conflict in situ, we employ metagenomics and metatranscriptomics to revisit a model ecosystem that has inspired many foundational discoveries in anaerobic ecology—methanogenic bioreactors. Through analysis of 17 anaerobic digesters, we recovered 1343 high-quality metagenome-assembled genomes and corresponding gene expression profiles for uncultured lineages spanning 66 phyla and reconstructed their metabolic capacities. We discovered that diverse uncultured populations can drive H_2_-sensitive metabolisms through (i) metabolic coupling with concurrent H_2_-tolerant catabolism, (ii) forgoing H_2_ generation in favor of interspecies transfer of formate and electrons (cytochrome- and pili-mediated) to avoid thermodynamic conflict, and (iii) integration of low-concentration O_2_ metabolism as an ancillary thermodynamics-enhancing electron sink. Archaeal populations support these processes through unique methanogenic metabolisms—highly favorable H_2_ oxidation driven by methyl-reducing methanogenesis and tripartite uptake of formate, electrons, and acetate.

**Conclusion:**

Integration of omics and eco-thermodynamics revealed overlooked behavior and interactions of uncultured organisms, including coupling favorable and unfavorable metabolisms, shifting from H_2_ to formate transfer, respiring low-concentration O_2_, performing direct interspecies electron transfer, and interacting with high H_2_-affinity methanogenesis. These findings shed light on how microorganisms overcome a critical obstacle in methanogenic carbon cycles we had hitherto disregarded and provide foundational insight into anaerobic microbial ecology.

Video Abstract

## Background

**Fig. 1:**
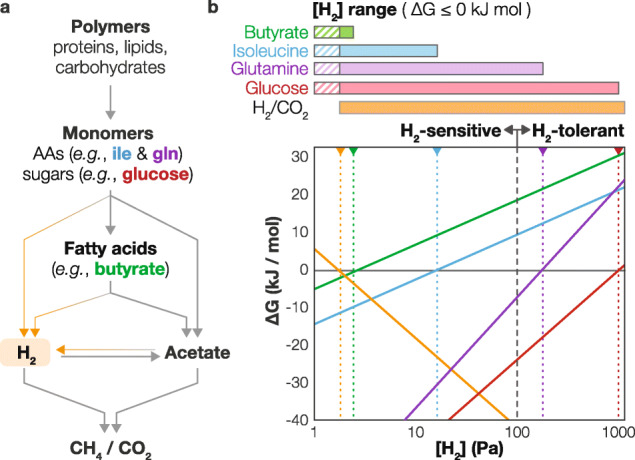
General scheme of methanogenic organic compound degradation and the “H_2_ conflict.” **a** Scheme for the degradation of organic macromolecules and the major intermediates, including AAs (blue or purple [see below]), sugars (red), FAs (green), and H_2_ (orange). **b** Gibbs free energy change for the degradation of representative AAs with low (isoleucine; blue) and high (glutamine; purple) calculated H_2_ tolerance, sugar (glucose), and fatty acid (FA) (butyrate) and H_2_-oxidizing CO_2_-reducing methanogenesis with varying H_2_ partial pressures. The vertical dotted lines indicate each pathway’s threshold H_2_ concentration at which ∆G becomes 0 kJ/mol. ∆G values are calculated as the ∆G_reaction_ + (mol ATP generated/mol reaction)*∆G_ATPsynthesis_ (see details below). The H_2_ partial pressure range at which each metabolism is thermodynamically favorable is shown at the top (horizontal bars with corresponding colors). Hydrogen partial pressures that overlap with those for H_2_-oxidizing CO_2_-reducing methanogenesis are indicated (solid colors) and would be permissive for that reaction. Metabolisms with [H_2_]_max_ less than 100 Pa and greater than 100 Pa are respectively defined as H_2_-sensitive and H_2_-tolerant. The following conditions were used for calculations—10 μM butyrate, 300 μM acetate, 0.1 μM amino acids and sugars, 1 mM NH_4_^+^, 50 mM HCO_3_^-^, 50 kPa CH_4_, pH of 7, and 37 °C. ∆G_ATPsynthesis_ is assumed to be 60 kJ/mol. For butyrate, isoleucine, glutamine, glucose, and H_2_/CO_2_ methanogenesis, ATP yields of 0.33, 1, 1.33, 4.67, and 0.2 were assumed. The ATP yields are calculated as follows: ATP_generated_ – ATP_consumed_ – x*(NADH_generated_ –NADH_consumed_) + x*(FdH_2generated_ – FdH_2consumed_) – 2x*(ETFH_2generated_ – ETFH_2consumed_) – 2x*(quinol_generated_ – quinol_consumed_), where *x* is the ATP synthase ATP:H^+^ ratio (assumed to be 1:3 for organotrophy and 1:5 for methanogenesis in this figure). Abbreviations: NADH—reduced nicotinamide adenine dinucleotide; FdH_2_—reduced ferredoxin; ETF—reduced electron transfer flavoprotein

Methanogenic bioprocesses are capable of converting municipal and industrial waste to methane and, thus, are paramount for achieving a sustainable environment [1, 2]. These processes have also served as model ecosystems throughout the history of anaerobic microbiology, including the discovery of syntrophic bacteria [3–5], isolation of model H_2_- [6] and acetate-utilizing [7–9] methane-generating archaea, and characterization of novel modes of bacteria-archaea symbiosis [10, 11]. Such pioneering studies generated our current understanding of how methanogenic microbial communities mineralize organic matter in both natural and engineered ecosystems (Fig. 1a)—(i) polymer hydrolysis to monomers, (ii) monomer (e.g., sugars and amino acids [AAs]) decomposition to H_2_, acetate, and other fatty acids (FAs; “acidogenesis”), (iii) FA degradation to H_2_ and acetate, (iv) interconversion of H_2_ and acetate (“acetogenesis”/syntrophic acetate oxidation), and (v) transformation of H_2_ and acetate to CH_4_ and CO_2_ (“methanogenesis”) [1, 12–15]. However, the majority of microbial populations in methanogenic bioprocesses/ecosystems has eluded cultivation and characterization [16, 17], suggesting we are far from fully comprehending the intricacies of the microbial ecology driving methanogenic decomposition. Uncovering the ecophysiology of the uncultured organisms, their ecological interactions, and the carbon and electron flow they create as a community is essential for advancing anaerobic microbiology, furthering our comprehension of the anaerobic sector of Earth’s biogeochemical cycles, and inspiring innovation in anaerobic biotechnology.

In the well-accepted scheme of methanogenic carbon-cycling, carbohydrate, AA, and FA degraders, all generate and transfer H_2_ to methanogens (Fig. 1a) due to the lack of favorable electron acceptors. This H_2_ transfer is a critical component of methanogenic decomposition as many processes cannot proceed without H_2_ being maintained at low concentrations, an interaction known as “syntrophy” [18]. However, the co-existence of the above diverse H_2_-generating processes is paradoxical. Many H_2_-generating metabolic processes are thermodynamically favorable and can produce H_2_ at high concentrations (H_2_-tolerant, HT, [H_2_]_max_ ≥ 100 Pa) (e.g*.*, 1020 Pa for glucose degradation; Fig. 1b and S1), whilst others can be inhibited by much lower H_2_ concentrations (H_2_-sensitive, HS, [H_2_]_max_ < < 100 Pa) (e.g., 2.8 Pa H_2_ for butyrate degradation; Fig. 1b and S1) [18–20]. (The concentration threshold was set at 100 Pa due to the large observed gap in H_2_ tolerances between 16 and 119 Pa; Fig. S1 and Table S1.) Although H_2_-scavenging methanogens can maintain low H_2_ concentrations to symbiotically support organisms performing HS metabolism (an interaction known as “syntrophy”), the high abundance and activity of organisms performing HT metabolism may generate high H_2_ concentrations above which HS metabolism cannot function. For example, the H_2_ concentration in co-cultures of H_2_-producing organotrophic bacteria and hydrogenotrophic methanogens can vary significantly depending on the substrates (1 ~ 2 Pa for fatty acids [21], ~ 20 Pa for aromatic compounds [22], ~ 60 Pa for select amino acids [23], > 700 Pa for lactate and ethanol [24]). Thus, in the presence of organisms performing HT metabolisms, partner methanogens may not maintain H_2_ concentrations sufficiently low for HS metabolisms. Moreover, anaerobic digester H_2_ concentrations can exceed the theoretical maximum H_2_ concentration threshold for many HS syntrophic metabolisms (up to 20 Pa [25–27]). Therefore, for HS and HT metabolisms to proceed concurrently, organisms performing HS metabolism must either spatially segregate based on thermodynamic properties or be able to transfer and/or dispose of electrons through alternative routes to circumvent thermodynamic inhibition. In the context of anaerobic bioreactors, spatial segregation has thus far only been observed in reactors that allow extensive biofilm formation (e.g., upflow anaerobic sludge blanket reactors) [28], suggesting the latter may be an especially important metabolic strategy in anaerobic digestion. This “hydrogen conflict” may be an overlooked component of the ecology in both natural and engineered methanogenic ecosystems. We suspect the absence of this selective pressure in conventional cultivation strategies could be a major factor contributing to why a significant proportion of the predominant microbial diversity in methanogenic ecosystems remains uncultured.

This thermodynamic paradox of HS and HT reactions co-occurring in proximity to each other is further exacerbated by the extremely low substrate concentrations available in situ. In many methanogenic engineered and natural ecosystems, the energy input is a complex mixture of macromolecules (i.e., detritus derived from dead bodies/cells of animals, plants, and microorganisms). Hydrolysis of detritus macromolecules releases diverse soluble monomers and oligomers that are subsequently absorbed and catabolized with little to no accumulation (10^-7^ to 10^-6^ M based on AA transporter *K*_*d*_ values [29]). Similarly, monomer-derived FAs only accumulate to micromolar levels [30, 31]. Such substrate concentrations, orders of magnitude lower than conventional cultivation media, can impede HS metabolism. For example, a three-order lower butyrate availability (e.g., 10 mM to 10 μM) can decrease the maximum tolerable H_2_ concentration for butyrate degradation by 30-fold (e.g., 88 Pa to 2.8 Pa; assuming conditions in Fig. 1b).

In the conventional scheme of methanogenic degradation, organotrophic metabolisms converge into a shared pool of H_2_, yet many metabolisms are only thermodynamically possible at low H_2_ concentrations and may be inhibited by activity of other concurrent H_2_-generating metabolisms. How organisms thrive under such thermodynamic restrictions remains unknown as most populations abundant in methanogenic ecosystems remain uncultured, possibly due to contrasting in situ and in vitro conditions. To characterize these organisms without cultivation, metagenomics and metatranscriptomics are effective tools that allow direct recovery of genomes (or metagenome-assembled genomes—MAGs) and gene expression profiles from the target ecosystem [32]. Although such “omics”-based methods predict rather than prove biological phenomena, rigorous analyses can provide valuable insight into potentially novel microbial processes taking place in situ. Moreover, given challenges associated with tracking the fate and turnover of H_2_ [33], a gene expression-based approach is one of the few strategies available for effectively tracing related behavior. In this study, we integrate omics analyses across multiple bioreactors, rigorous anaerobiosis-tailored metabolic reconstruction, and thermodynamics to unveil the ecophysiology and metabolic strategies of uncultured microbial populations tailored to driving thermodynamically sensitive metabolic processes in a model methanogenic ecosystem, anaerobic digesters.

## Results and discussion

### Metagenomic and metabolic reconstruction

**Fig. 2:**
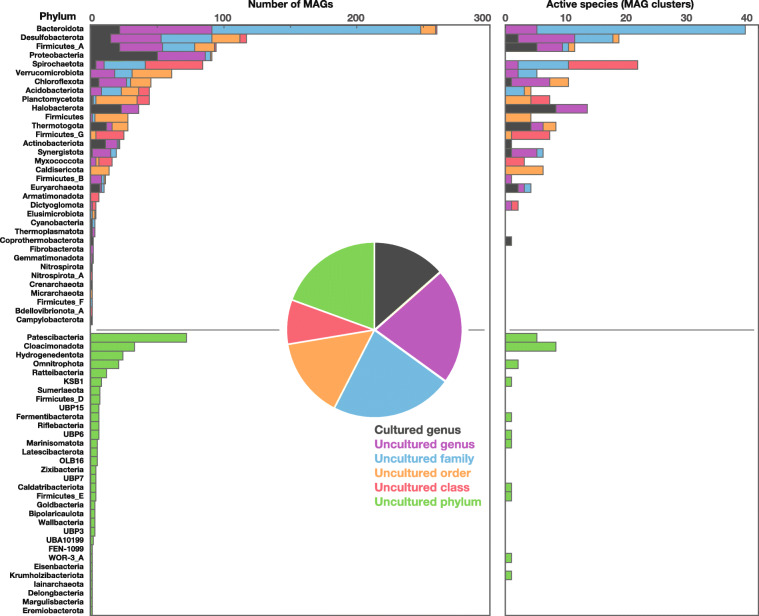
Phylogenetic distribution of MAGs recovered and species (MAG clusters) associated with a high metatranscriptome-based activity. The phylogenetic classification was determined using GTDBtk (left). The number of MAGs associated with a cultured genus or uncultured lineages (at different taxonomic levels) is shown (right). Bacterial and archaeal species respectively associated with metatranscriptome-based activities ≥ 0.4% or ≥ 0.3% of the mapped transcriptomes in at least one reactor are shown

Metagenomics analysis of 17 full-scale anaerobic digesters treating wastewater sludge yielded 1343 metagenome-assembled genomes (MAGs) that meet the quality criteria previously proposed (CheckM-estimated completeness—contamination > 50 [34, 35]; for *Ca.* Patescibacteria ≥ 60% and ≤ 5% was used given the inherently low estimated completeness for members of this phylum). These MAGs spanned 66 phyla, as predicted by GTDBtk [35] (Fig. 2 and S2, Tables S2 and S3). The MAGs retained had estimated completeness and contamination ≥ 85% and ≤ 7.5%, respectively (as predicted by CheckM), except for MAGs affiliated with *Ca.* Patescibacteria (≥ 60% and ≤ 5%). Out of the obtained MAGs, only 181 were assignable to cultured genera and the remaining belonged to various uncultured genus- (289 MAGs), family- (303), order- (199), class- (110), and phylum-level lineages (261) (Fig. 2). MAGs were clustered into 896 species using Mash [36], based on a pairwise mutation distance of ≤ 0.05 (or ≥ 95% average nucleotide identity), which roughly equates to a 70% DNA-DNA reassociation value that has been proposed as a genome-based species definition [37]. Based on metatranscriptomes recovered from 9 full-scale anaerobic digesters (all in triplicate; Tables S2 and S3), 176 bacterial species and 16 archaeal species each had transcripts representing ≥ 0.4% and ≥ 0.3% of the mapped transcriptomic reads, respectively, in at least one digester (Table S4 and S5). These species with high relative activity are herein referred to as “active” species. Much of the remaining species belong to taxa associated with residual populations (primarily non-*Deltaproteobacteria Proteobacteria* classes and non*-Bacteroidia Bacteroidetes* classes) [38, 39] carried in as waste from upstream aerobic bioprocesses (Fig. S3). Thus, species associated with the above taxa were excluded from following analyses. The “active” species spanned 20 cultured and 11 uncultured phyla, of which Bacteroidota (previously known as *Bacteroidetes*), Desulfobacterota (*Deltaproteobacteria*), Firmicutes_A (*Firmicutes*), Spirochaetota (*Spirochaetes*), and Halobacterota (*Euryarchaeota*) were most frequently detected (Fig. 2). These taxonomic groups and their nomenclature follow the genome-based phylogeny recently proposed [35]. Among these, 91.5% and 41.2% of the active bacterial and archaeal species, respectively, belonged to uncultured lineages, clearly implying large knowledge gaps in how bacteria and archaea mineralize organics in situ (Table S4).

To accurately reconstruct the metabolic behavior of individual species, we annotate metabolic pathways with strict criteria by taking advantage of the thermodynamic and energetic restrictions of anaerobic life. Due to the cavernous gap in electron acceptor redox potentials (e.g., O_2_ to H_2_O [E°’ of 1.23 V] vs H^+^ to H_2_ [E°’ of − 0.42 V]), aerobic degradation is highly exergonic and massive energy recovery occurs from O_2_ reduction (e.g*.*, ~ 32 ATP per glucose), while anaerobic degradation is much more thermodynamically limited and often requires energy investment for disposing electrons. Moreover, certain anaerobic metabolisms can become endergonic (i.e*.*, ∆G > 0 kJ mol^-1^) with only slight byproduct (e.g*.*, H_2_) accumulation and require intimate cross-feeding with byproduct-consuming partners, a symbiosis known as syntrophy [3]. To thrive at this thermodynamic edge of life, anaerobes must employ unique metabolic strategies for coupling substrate oxidation with electron disposal and optimizing energy input and recovery during this process [40–42]. Pioneering efforts in isolating and characterizing syntrophic metabolizers and their enzymes was paramount for obtaining this foundational knowledge [4, 5, 43–45]. Capitalizing on these unique insights into syntrophic metabolism, we identified for each active species metabolic pathways that (i) have electron transfer enzymes that account for all predicted oxidative and reductive reactions, (ii) provided net positive energy conservation either by ATP synthesis and/or by the generation of an ion motive force, (iii) are exergonic in situ, and (iv) have all necessary genes highly expressed (see details in methods section; Fig. S4 as an example; and supplementary tables for a summary of capacities [Table S4], summary of H_2_/formate-generating electron transfer capacities [Table S6], summary of metabolic behavior across digesters [Table S7–S9], metabolic behavior in individual reactors [Table S10–S18; and Table S19 for all collected together]). The maximum metabolic capacity observed within a species cluster and the total metabolic capacity of that cluster were similar (Table S4 and Fig. S5), suggesting consistent ecological roles across digesters.

**Fig. 3:**
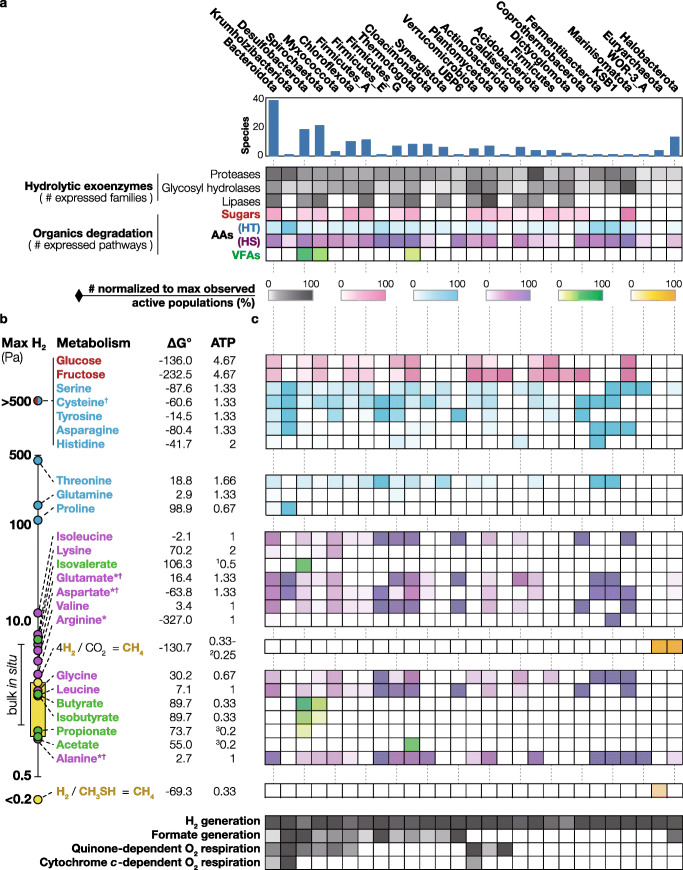
Phylum-level overall metabolic activities, the thermodynamics-based H_2_ thresholds of the activities, and expression of individual pathways. **a** For each phylum, the number of species clusters, average number of protease, glycosyl hydrolase, and lipase families expressed across species are shown (normalized to maximum observed average among phyla). Likewise, the average number of sugar-, AA-, and FA-degradation pathways expressed across species is shown. AA degradation pathways are split into those that are H_2_-tolerant (HT) and H_2_-sensitive (HS) based on panel b. **b** The maximum H_2_ concentration that each degradation pathway can tolerate is shown (i.e., ∆G_reaction_ + x*∆G_ATPsynthesis_ = 0, where *x* is the amount of ATP synthesized per substrate degraded). The ATP yield for each pathway was based on the sum of (i) the ATP consumption/generation in the main carbon transformation pathway and (ii) vectorial H^+^ translocation associated with membrane-based electron transfer (e.g., Rnf, Hyb, Fdn), assuming the shortest electron flow route from substrate oxidation to H_2_/formate generation that involves electron bifurcation and reverse electron transport where possible; all of this was based on pathways that were observed to be expressed in this study. Reactions that would either lose much energy as heat (e.g., cytosolic Fd_red_-oxidizing H_2_ generation) or require energy input under in situ conditions (e.g., cytosolic NADH-oxidizing H_2_ generation) were not considered. For substrates whose degradation proceeds through pyruvate or acetyl-CoA, maximum H_2_ concentrations for oxidation to acetate are shown (see Supplementary Table S1 for a list of reactions). Note that fermentation pathways (e.g., acetyl-CoA reduction to butyrate) would increase the maximum H_2_ but reduce ATP yield. The Gibbs free energy yield at standard conditions and pH 7 (∆G°’) and estimated ATP yields are also shown. See Fig. 1 for details for calculating ATP yield and maximum tolerable H_2_ concentration. For each pathway, ∆G_reaction_ was calculated assuming 300 μM acetate, 10 μM for other FAs, 1 mM NH_4_^+^, 50 kPa CH_4_, 50 mM HCO_3_^-^, 37 °C, 3.9 × 10^-4^ atm H_2_S, and 0.1 μM for all other compounds. ∆G_ATPsynthesis_ was assumed to be 60 kJ/mol. *Although more exergonic alternative pathways exist for these HS AA degradation pathways (e.g., through butyrate fermentation), species only expressing the HS pathway(s) were identified in situ, indicating that HS metabolism of these AAs is relevant in situ. ^1^For isovalerate degradation, an ATP synthase ATP:H^+^ ratio of 1:4 was assumed. ^2^For H_2_-oxidizing CO_2_-reducing methanogenesis, two H_2_ concentrations for two ATP yields assuming different ATP synthase ATP:H^+^ ratios. ^3^For propionate and acetate degradation, an ATP synthase ATP:H^+^ ratio of 1:5 was assumed. ^†^Pathways whose directionality cannot be determined by sequence data alone. **c** For each phylum, the percentage of species expressing individual degradation pathways are shown

Metabolic reconstruction and metatranscriptome mapping of the 192 species clusters revealed that the phyla contributing most to degradation of polymers (i.e*.*, expressing multiple extracellular proteases, glycosyl hydrolases, and lipases) were Bacteroidota (42 families on average), Verrucomicrobiota (49), Planctomycetota (38), Acidobacteriota (44), and Marinisomatota (68) (Table S20). Phyla contributing most to the degradation of monomers (i.e., expressing multiple sugar and AA degradation pathways) were Bacteroidota (3.6 and 6.9 types of sugar and AA degradation pathways, respectively, on average), Firmicutes_E (0 and 9), Thermotogota (3.3 and 7.6), KSB1 (0 and 9), and Marinisomatota (6 and 8) (Fig. 3a). Most other phyla also contributed polymer hydrolysis and the subsequent degradation of sugars and AAs but expressed fewer polymer- and monomer-degrading pathways (≤ 36 hydrolase families and ≤ 7 pathways for sugar and AA degradation). The above metabolisms generate FA byproducts such as acetate, propionate, butyrate, isobutyrate, 2-methylbutyrate, and isovalerate whose degradation is highly thermodynamically challenging [17, 19, 46]. Of the 29 active bacterial phyla, only three expressed genes for the oxidation of these FA—Desulfobacterota (12 species), Spirochaetota (3), and Thermotogota (3). To accurately predict capacities to degrade different FAs, each FA degradation pathway (methylmalonyl-CoA pathway [propionate], beta-oxidation [butyrate], mutase + beta-oxidation [isobutyrate], carboxylation + lyase + beta-oxidation [isovalerate]), and hydrogenases (e.g*.*, FeFe and bidirectional NiFe hydrogenases), formate dehydrogenases (e.g*.*, Fdh-H and Fdh-N type), electron transfer modules (e.g*.*, Rnf), and energy conservation that complement each other and allow net energy recovery (e.g*.*, transcarboxylation for propionate and ETF dehydrogenase for C4 and C5 FAs) were identified. For Desulfobacterota, uncultured members of a Syntrophales family contributed to butyrate, isobutyrate, and isovalerate degradation; the Desulfomonalia order contributed to butyrate and isobutyrate degradation, and a Syntrophobacteraceae genus contributed to propionate degradation. Many Desulfobacterota species also concurrently expressed genes for the degradation of multiple FAs (up to three substrates), a feature that has been unobserved and untested in anaerobic FA-degrading isolates [19]. Members of an uncultured Spirochaetota class expressed genes for butyrate and isobutyrate degradation. Thermotogota species belonging to an uncultured Thermotogae order was predicted to perform acetate degradation. Members of Halobacterota (i.e., *Methanothrix*, also formerly known as *Methanosaeta*) and other Halobacterota/Euryarchaeota (e.g., *Methanoculleus*, *Methanospirillum*, *Methanothermobacter*, and uncultured *Methanomicrobiaceae*), respectively, contribute to the degradation of terminal end products, acetate, H_2_, and formate.

### Thermodynamic conundrum

The co-existence of the above processes is puzzling in terms of thermodynamics. Most forms of organotrophy in methanogenic ecosystems are presumed to dispose electrons by reducing H^+^ to H_2_. However, some types of organotrophy can produce H_2_ to levels that can thermodynamically inhibit other types if H_2_ accumulates to sufficient levels. The question then is how does H_2_-mediated interspecies electron transfer from organotrophic bacteria to methanogenic archaea, which is a core process in methanogenic ecosystems, proceed in these ecosystems? Based on our calculations, the maximum tolerable H_2_ concentration varies significantly among substrates and pathways involved (Fig. 3b). Degradation of sugars and many AAs is highly exergonic and HT, while the degradation of FAs and certain AAs is HS and may require exceptionally low H_2_ concentrations (≤ 16 Pa). Despite the rapid H_2_ consumption by partner methanogens, the high activity and abundance of organisms performing HT metabolism (44 ~ 70% of mapped metatranscriptomes) may generate localized high H_2_ concentrations that can inhibit organisms performing HS metabolism. Moreover, the estimated maximum H_2_ concentration thresholds for HS AA degradation (1.1 ~ 10.3 Pa H_2_) and FA degradation (1.2 ~ 2.8 Pa H_2_) are very close to the minimum hydrogen threshold that conventional H_2_-utilizing CO_2_-reducing methanogenesis can use (1.7 Pa H_2_), which is often lower than bulk H_2_ concentrations detected in reactors (< 10 Pa) [25, 47]. Thus, we expect that the species performing HS metabolism may have unique strategies to circumvent these thermodynamic obstacles.

### Coupling H_2_-tolerant (HT) and H_2_-sensitive (HS) metabolisms

**Fig. 4:**
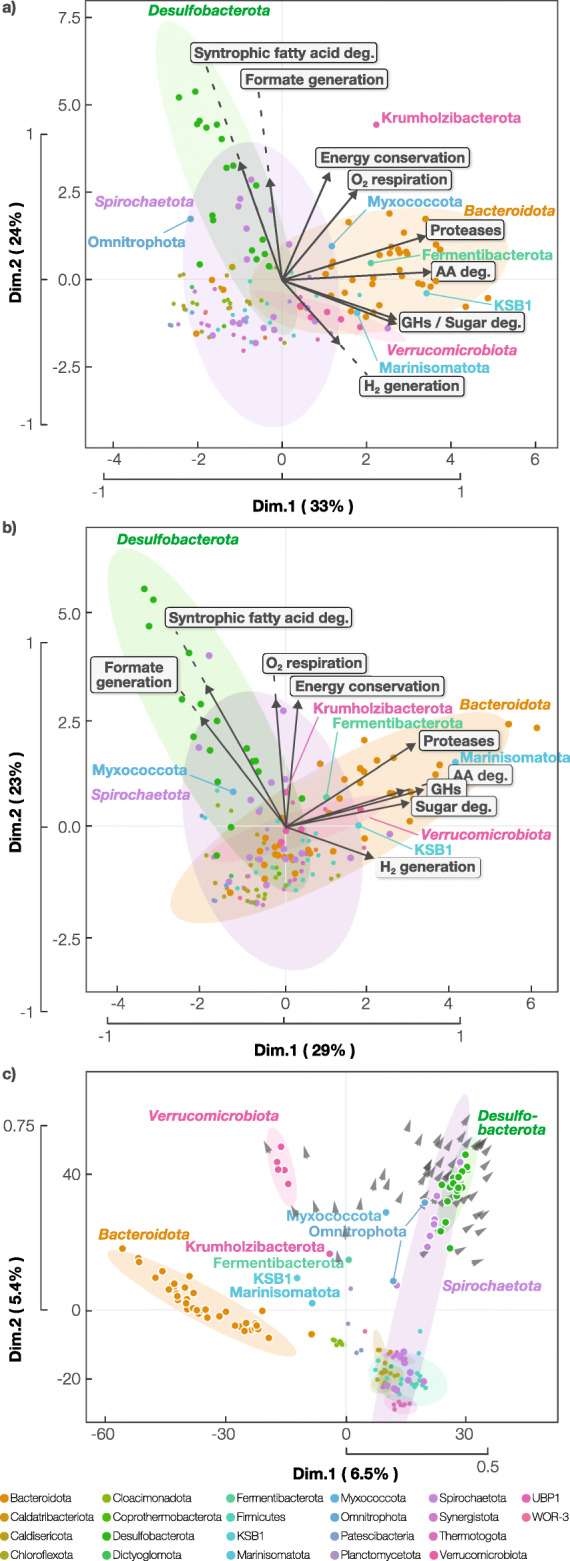
Principal component analysis (PCA) of **a** metabolic capacities, **b** expressed pathways, and **c** individual genes/functions for active species. **a** PCA of active species and their metabolic capacities: proteases and glycosylhydrolases (GHs) as the number of families encoded in the genome; FA, AA, and sugar degradation as the number of pathways encoded in the genome; electron transfer/energy conservation pathways (i.e., Rnf, Nfn, Fix, Efd, and FloxHdr) as the number of pathways encoded in the genome; H_2_ and formate generation as presence/absence; and cytochrome bd oxidase-mediated O_2_ respiration as presence/absence. Individual species (points) and metabolic capacities (vectors) are shown. Confidence ellipses (95%) are shown for MAGs belonging to specific phyla. **b** PCA of active species and the metabolic behavior they expressed: proteases and glycosylhydrolases (GHs) as the number of families expressed in at least one reactor; FA, AA, and sugar degradation as the number of complete pathways expressed in at least one reactor; electron transfer/energy conservation pathways (i.e., Rnf, Nfn, Fix, Efd, and FloxHdr) as the number of pathways expressed in at least one reactor; H_2_ and formate generation as the highest hydrogenase/formatted dehydrogenase subunit expression level (calculated as RPKM normalized to specie’s non-zero median expression level); and cytochrome bd oxidase-mediated O_2_ respiration as the highest oxidase subunit expression level. Individual species (points) and metabolic capacities (vectors) are shown. **c** PCA of active species and their functional profiles predicted through eggNOG. Functions that are detected at a significantly higher frequency in Desulfobacterota and Spirochaetota than other phyla (*p* < 0.05) are shown as vectors. The functions associated with these vectors are shown in Table S16

To identify ancillary metabolic pathways supporting HS metabolisms in situ, we compared catabolic capacities across the 192 high-activity species. Pearson correlation revealed correspondence between the number of HS AA metabolisms per species cluster and the number of hydrolytic exoenzyme families (glycosylhydrolases [*p* = 6.8 × 10^-5^] and proteases [*p* = 1.3 × 10^-20^]), pathways for HT AA metabolism (*p* = 7.4 × 10^-76^), sugar degradation pathways (*p* = 4.5 × 10^-6^), and types of both [FeFe] and [NiFe] hydrogenases (*p* = 2.3 × 10^-11^ and 0.033, respectively) (see Table S4 for categories and values used for all Pearson correlation calculations). This suggests an interaction between hydrolysis of a wide range of polymers, simultaneous catabolism of multiple types of polymer-derived monomers, and diverse H_2_ generation pathways. Comparison of phyla showed that Bacteroidota encoded significantly more pathways for HS and HT AA metabolism (*p* = 0.016 and 0.018 respectively; Student’s *t* test), glycolsylhydrolases (0.023), and proteases (0.003) than other phyla. Correlation analyses were not possible for other phyla with fewer species, but the principal component analysis also suggested a qualitative association of Fermentibacterota, Marinisomatota, Verrucomicrobiota, and KSB1 with these features (Fig. 4a, b). We found that many species of these phyla (25 out of 38 species in Bacteroidota, 1 out of 1 in Fermentibacterota, 1 out of 1 in Marinisomatota, 1 out of 5 in Verrucomicrobiota, and 1 out of 1 in KSB1) expressed genes for both HS and HT metabolism of AAs (e.g., HS and HT AA degradation with H_2_ formation) (see Tables S7-S9 for overviews and S10-S18 for individual reactors). Of these, 24 Bacteroidota, 1 Fermentibacterota, 1 Verrucomicrobiota, and 1 KSB1 species were confirmed to consistently perform the above metabolism based on the following criteria: expressing the complete metabolic pathway(s) in at least 50% of the studied reactors where this species comprised ≥ 0.05% of the mapped metatranscriptome (herein referred to as ECM50 species; Table S8). We detected HS sensitive pathways for lysine (Bacteroidota), isoleucine/leucine/valine (Bacteroidota and Marinisomatota), arginine (Bacteroidota and KSB1), glutamate (Bacteroidota, KSB1, and Fermentibacterota), glycine (all five phyla), and alanine (all five phyla). (Note that, with rigorous annotation as outlined in the methods [see Fig. S4 as an example], we can determine the directionality of most AA metabolism pathways, exceptions being alanine, cysteine, glutamate, and aspartate metabolism [Table S1]). Although HS metabolism would be thermodynamically inhibited by H_2_ generated from HT degradative processes in proximal cells or in the same cell, HS, and HT metabolism pathways intersect at shared metabolic intermediates (e.g., NAD[H], NADP[H], and/or ferredoxin) that could potentially be coupled enzymatically to provide for thermodynamically favorable redox reactions.

Though the hydrolytic organisms could theoretically focus on performing HT metabolism, we suspect, based on our analysis of the pathways present in diverse metagenomes, that these organisms degrade wide ranges of substrates (both HS and HT AA metabolism) to maximize energy recovery from the heterogeneous pool of monomers generated from polymer hydrolysis, thereby compensating for the high energy cost associated with producing extracellular hydrolytic enzymes [48]. It is important to note that HS and HT AA metabolism generally have similar ATP yields despite thermodynamic differences in substrate degradation. We suspect this energy compensation is important for the above phyla as they express a wide range of hydrolytic enzymes. For protein hydrolysis, many species clusters associated with the above phyla were in the top 30% of all active species for the average number of protease families expressed when active (i.e., in reactors they displayed ≥ 0.5% metatranscriptome-based activity) (> 7.8 families)—35 Bacteroidota (34 of which were ECM50 species), 1 Fermentibacterota (1 ECM50 species), 1 Marinisomatota (1 ECM50 species), 4 Verrucomicrobiota (4 ECM50 species), and 1 KSB1 (1 ECM50 species) respectively) (Table S19). Similarly, 21 (21 ECM50 species), 0, 1 (1), 5 (5), and 1 (1) species cluster(s) respectively for carbohydrate hydrolysis (> 7.6 glycosylhydrolase families) and 5 (5 ECM50), 0, 1 (1), 1 (1), and 0 species cluster(s) respectively for lipid hydrolysis (> 1.2 lipase families). In addition, Pearson correlation revealed an association between the numbers of families encoded for each exoenzyme type (all *p* ≤ 3.0 × 10^-7^). Thus, these versatile anaerobes hydrolyze a broad range of polymers, generate diverse monomers in the process, and use thermodynamically favorable monomer degradation reactions to drive the concomitant degradation of other monomers whose degradation would be otherwise thermodynamically unfavorable. Nearly all species (96.7% or 88 out of 91) that were predicted to perform HS metabolism couple HS and HT AA degradation in at least one reactor (Table S9), suggesting this is the predominant strategy to accomplish HS AA degradation.

### Shifting to interspecies formate transfer

Unlike polymer/monomer catabolism, the number of syntrophic FA degradation pathways encoded in a species cluster had a negative correlation with the number of [FeFe] hydrogenases (Pearson correlation *p* = 0.044; Fig. 4a, b). This suggests that the FA-degrading syntrophic metabolizers likely employ an alternative route for the re-oxidation of their reduced carriers. While H_2_ exchange is the most well-recognized mode of interspecies electron transfer, CO_2_-reducing formate generation also serves as an important mechanism for electron disposal and transfer [33, 42, 44, 49]. FA catabolism indeed had a unique positive correlation with both Fdh-H type (cytosolic) and Fdh-N type (membrane-associated) formate dehydrogenases (Pearson correlation *p* = 2.1 × 10^-12^ and 1.5 × 10^-35^) not observed for AA and sugar metabolism. Nearly all Desulfobacterota species (12 total across uncultured Desulfomonalia order UBA1602, Syntrophales families UBA8958 and UBA2192, and Smithellaceae) actively performing syntrophic FA metabolism in at least one reactor expressed genes for CO_2_-reducing formate generation (11 ECM50 species out of 12 total or 91.7%) and, of these, most only expressed genes for formate generation and not for H_2_ generation (82.0% ECM50 species; Table S9). In agreement, Desulfobacterota had significantly higher numbers of FA degradation (Student’s *t* test *p* = 0.045) and Fdh-H/Fdh-N type formate dehydrogenases (*p* = 0.044 and 0.032) compared to other phyla. Most Spirochaeotota (uncultured class UBA4802) populations expressing syntrophic butyrate degradation also expressed genes for formate generation (two out of three FA-degrading Spirochaeotota species ECM50). We also observed a correlation between FA metabolism and Fdh-N type formate dehydrogenases with the number of intracellular energy-conserving electron transport enzyme complexes (see Table S6 for list) (Pearson correlation *p* = 6.4 × 10^-5^ and 2.7 × 10^-4^, respectively), indicating the importance of possessing multiple energy conservation routes for syntrophic FA degradation. Through comparing the presence/absence of individual functions (based on automatic emapper-based annotations) across all active species clusters (Fig. 4c and Table S20), we also identified correlation (Student’s *t* test; *p* < 0.05) in Desulfobacterota and Spirochaetota between the FA-degrading enzyme acyl-CoA dehydrogenase, electron transfer flavoprotein:quinone oxidoreductase, and formate dehydrogenases Fdh-H and Fdh-N, which plots out the route of electron flow for the most thermodynamically difficult redox reaction involved in syntrophic FA metabolism—the generation of formate or H_2_ from electrons derived from acyl-CoA oxidation. Earlier proteomic studies implied these enzyme systems for H_2_ or formate production from electrons derived from acyl-CoA oxidation in *Syntrophomonas wolfei* [45, 50]. The finding that this same enzyme system is used in diverse bacteria suggests that this may be the common mechanism for the difficult redox reaction. Remarkably, many populations lacked hydrogenases (53.3% or 8 out of 15 species; see Table S6 for hydrogenases surveyed). This observation is in stark contrast with what is known about isolated syntrophic organisms, which all possess hydrogenases and employ H_2_ as an interspecies electron carrier [19, 51]. However, further proteomic studies are necessary to verify the absence of hydrogenases in these novel syntrophic populations. We also identified three putative syntrophic acetate-degrading species clusters in Thermotogota (*Pseudothermotoga* and an uncultured Thermotogae order) expressing a previously proposed glycine-mediated acetate degradation pathway (two acetate-degrading Thermotogota ECM50 species) [17]. Two coupled this with formate generation (no H_2_ generation) in at least one reactor (one out of two acetate-degrading Thermotogota species ECM50).

Unlike hitherto characterized syntrophs, which are cultured in the absence of other H_2_-generating processes (i.e., only one substrate in the culture medium), these newly discovered uncultured organisms may thrive in the presence of highly thermodynamically favorable H_2_-generating processes. We propose that organisms that perform HS FA catabolism avoid thermodynamic conflict with those that use HT-catabolism by completely or partially forgoing H_2_ generation and relying on formate transfer to efficiently transport electrons to physically distant metabolic partners [33, 49, 50]. In contrast with H_2_, formate concentrations are unlikely to accumulate locally in situ as formate-producing activity is absent or low in most polymer/monomer-degrading species (i.e., most of the active community) based on our analyses and formate has a higher diffusion rate than H_2_ [33, 49]. Although formate is challenging to detect in anaerobic digesters, it is estimated to be at concentrations around 2.5 μM (equivalent to 4.5 Pa H_2_ at 37 °C, pH 7, and 50 mM HCO_3_^-^) [33]. Moreover, formate transfer would allow FA degraders to recover additional energy via ion-translocating, formate transporters [42] (expressed by 88.9% of FA-degrading species).

Though uncommon, 18 species were found to couple formate generation with HS AA catabolism (Table S7-S9; alanine, glycine, glutamate, isoleucine, leucine, lysine, or valine). These organisms span eight phyla and uncultured lineages that have never been reported to be capable of syntrophic interactions: candidate phylum UBP6, uncultured phylum Krumholtzibacteriota, uncultured phylum Cloacimonadota, Bacteroidota (uncultured *Bacteroidales* family), Chloroflexota (unc. *Anaerolineaceae*), Desulfobacterota (unc. Syntrophorhabdaceae), Firmicutes_E (unc. class DTU015), Firmicutes_G (unc. *Limnochordia* order DTU010), Myxococcota (unc. class XYA12-FULL-58-9), Spirochaetota (unc. *Treponematales* family), and Thermotogota (unc. *Thermotogae* order). Given the thermodynamic sensitivity of the aforementioned AA degradations, these species likely rely on formate generation for the same reasons that the syntrophic FA degraders do. Although we cannot conclude syntrophic capabilities without cultivation, we found that the above species encode enzymes involved in supporting thermodynamically challenging reactions and metabolism: reverse electron transport (NADH:Fd oxidoreductase Rnf [UBP6, Krumholtzibacteriota, Cloacimonadota, Bacteroidota, Firmicutes_E, and Firmicutes_G]) and electron bifurcation (NAD-dependenet Fd:NADP oxidoreductase Nfn [UBP6, Bacteroidota, Desulfobacterota, Firmicutes_E, Spirochaetota, and Thermotogota species]). We also identified several syntroph-associated enzymes in the species’ genomes: monomeric formate dehydrogenase [42] (UBP6, Desulfobacterota, and Spirochaetota), electron transfer flavoprotein dehydrogenases Fix [51] or Efd [41] (Krumholtzibacteriota, Bacteroidota, Desulfobacterota, and Myxococcota), and uncharacterized syntroph-associated redox complex Flox-Hdr [52, 53] (Krumholtzibacteriota and Desulfobacterota). In addition, of the 18 identified formate-generating HS AA-degrading species, 7 (4 ECM50) did not couple HS metabolism with HT AA degradation in at least one reactor (UBP6, Chloroflexota, Cloacimonadota, Desulfobacterota, and Thermotogota), suggesting the need for formate-mediated syntrophic interaction to complete HS AA degradation

### Aerobic respiration by obligate anaerobes

Beyond the coupling of HS metabolism with HT metabolism or formate generation, we found a positive correlation between the number of HS AA and FA metabolism pathways with the presence of a cytochrome bd oxidase (Pearson correlation *p* = 4.3 × 10^-4^ and 2.3 × 10^-8^), a terminal oxidase for aerobic respiration (Fig. 4a, b). The transcription of these genes was detected in at least one reactor for 36.0% of species actively expressing HS AA degradation belonging to the five versatile hydrolytic phyla reported above (27.8% ECM50 species) and 75.0% of the Desulfobacterota and Spirochaetota species expressing syntrophic formate/H_2_-generating FA degradation (58.3% ECM50 species) (Table S7-S9). These organisms possess many O_2_-sensitive enzymes (e.g., pyruvate:ferredoxin oxidoreductase, 2-oxo-glutartate:ferredoxin oxidoreductase, formate dehydrogenases, and FeFe hydrogenases) and lack central O_2_-tolerant enzymes (e.g., pyruvate dehydrogenase and 2-oxoglutarate dehydrogenase), indicating that the organisms are strictly anaerobic and not facultatively aerobic. The association of heme biosynthesis genes (hemACL) with Desulfobacterota and Spirochaetota was also observed (Student’s *t* test *p* < 0.05; Fig. 4c), supporting the functionality of cytochrome bd oxidase. Although the anaerobic digestion ecosystem is considered to be strictly anaerobic, minute amounts of O_2_ can enter the system through the influent wastewater [54–56]. This is analogous to gas or water percolation from an aerobic zone to a neighboring anaerobic zone in natural ecosystems. Moreover, cytochrome bd oxidase can function even at nanomolar concentrations of O_2_ [57]. Using this low-concentration O_2_ as an alternative electron disposal route can reduce the dependence on H_2_ or formate production, which is thermodynamically sensitive to the accumulation of these byproducts and increases the thermodynamic favorability of their overall catabolism. For example, for butyrate oxidation in the presence of nanomolar levels of O_2_, redirecting 1% of the electrons towards O_2_ respiration can double H_2_ tolerance ([H_2_]_max_; from 2.7 to 5.6 Pa) and increase the thermodynamic favorability by 20% (∆G of − 13.3 to − 15.9 kJ/mol, assuming 10 Pa H_2_, 50 nM O_2_, and other conditions used in Fig. 3b). Moreover, the terminal oxidase can increase tolerance to oxidative stress by consuming O_2_. Indeed, previous studies have demonstrated that a strictly anaerobic organism can tolerate and benefit from nanomolar concentrations of O_2_ [58] and anaerobic digestion can benefit from controlled microaeration [56, 59]. Thus, organisms encountering kinetic and thermodynamic bottlenecks (i.e., hydrolysis and HS AA/FA degradation) of methanogenic organic matter mineralization may depend on O_2_ for optimal activity.

### New routes of electron flow in methanogens

To better understand interspecies electron transfer, we investigated the metabolic behavior of methanogenic archaea. As expected, most Euryarchaeota and Halobacterota expressed H_2_- and/or formate-driven CO_2_-reducing methanogenesis genes, syntrophically supporting electron disposal of organotrophic activity (Table S3). We also discovered high activity (gene expression) in *Ca.* Methanofastidiosa (previously known as class WSA2), an archaeon previously proposed to utilize methylated thiols as a carbon source for methanogenesis rather than CO_2_ [60]. Metatranscriptomics provided further evidence that *Ca.* Methanofastidiosum indeed performs H_2_-oxidation coupled to methylated thiol-reduction to methane in situ (all methyl-reducing Methanofastidiosum were ECM50 species) (Table S7). Based on thermodynamics, such methanogens can theoretically tolerate much lower H_2_ concentrations than those that use conventional H_2_/CO_2_ methanogenesis (0.1 Pa versus 1.7 Pa, respectively; assuming conditions in Fig. 3). This would mean that in the presence of methylated thiols (generated from the degradation of methylated compounds such as methionine), *Ca*. Methanofastidiosum can pull H_2_ concentrations to much lower levels than conventional methanogens and more effectively support H_2_ generation from HS metabolism. Thus, methylated compounds likely play an important role in overcoming thermodynamically challenging metabolisms in anaerobic digestion and other methanogenic ecosystems.

Although interspecies electron transfer in methanogenic ecosystems is often simplified as H_2_ exchange, such microbial interactions are clearly more complex. In addition to the exchange of metabolites such as H_2_ or formate, microorganisms can also directly transfer electrons to each other, a process called direct interspecies electron transfer (DIET) [11]. Yet, the prevalence and importance of DIET in anaerobic digestion are unclear. Among methanogens detected in situ, *Methanothrix* is the only lineage known to be capable of utilizing extracellular electrons to drive CO_2_-reducing methanogenesis [11], although it is most well known for its capacity to use acetate for methanogenesis. We identified three *Methanothrix* species expressing DIET-driven CO_2_ reduction and acetoclastic methanogenic pathways (all acetate-degrading *Methanothrix* were ECM50 species; Table S7-S9), indicating the presence of “exoelectrogenic” organisms in situ. Inspection of the transcriptomes revealed that 13 and 18 bacterial phyla may perform DIET respectively through multiheme c-type cytochromes (including members of uncultured phyla Omnitrophota, KSB1, and Krumholzibacterota) and conductive pili (including members of uncultured phyla Cloacimonadota, Omnitrophota, Patescibacteria, Krumholzibacterota, and WOR-3). Expression of multiheme c-type cytochromes was observed for Desulfobacterota and Spirochaeota performing syntrophic FA degradation (53.0% of FA-degrading Desulfobacterota and Spirochaetota species; 46.7% ECM50 species) and versatile polymer/monomer-degrading Bacteroidota, Verrucomicrobiota, and KSB1 (54.2%; 40.0% ECM50 species). For conductive pili, we found positive correlation for the presence of conductive pili with syntrophic FA degradation (Pearson correlation *p* = 8.0 × 10^-7^) and other capacities associated with in situ FA degraders (Fdh-N type formate dehydrogenase [*p* = 1.9 × 10^-8^], electron transfer complexes [*p* = 2.3 × 10^-4^], and cytochrome bd oxidase [*p* = 5.2 × 10^-3^]), while no correlation was observed with hydrolytic enzymes and AA/sugar degradation. We further confirmed that many FA-degrading Desulfobacterota and Spirochaetota species express conductive pili (60.0%; 46.7% ECM50 species), but only three populations of the versatile hydrolytic HS/HT AA degraders expressed putative conductive pili in at least one reactor (5.7% ECM50 species). Diverse phyla and niches likely take advantage of DIET because exoelectrogenic metabolism can theoretically much more thermodynamically favorable than H_2_ generation due to the high reduction potential of c-type cytochromes (E°’ of − 220 to + 180 mV [61]). The reason for the difference in the distribution of multi-heme cytochromes (all studied niches) and putative conductive pili (preferentially found in syntrophic FA degraders) remains unclear. Though the necessary physical proximity between syntrophs and electron-accepting partners may allow more opportunities for conductive pili to transfer electrons, further investigation is required. In total, hydrolysis, monomer degradation, and FA degradation by uncultured organisms across 20 phyla may rely on *Methanothrix* species for H_2_-independent extracellular electron transfer, though different niches may use different routes.

Further inspection of the transcriptomes revealed the possible involvement of *Methanothrix* in formate degradation. Although the ability of *Methanothrix* to degrade formate has been controversial [7, 62, 63], we detected consistent expression of a formate dehydrogenase complex in two out of three *Methanothrix* species in all reactors they were active (Table S7-S9). Based on gene organization of the formate dehydrogenase in the most active *Methanothrix* species JPASx098 (fdhA with hdrABC and ferredoxins; ≥ 99% similarity to *M. soehngenii* genes MCON_3277-83), *Methanothrix* may oxidize formate and funnel electrons into methanogenesis (via HS-CoM/HS-CoB and ferredoxin). We suspect that *Methanothrix* primarily performs acetate-driven methanogenesis but, in parallel, can uptake formate and electrons from extracellular pili and cytochromes to drive CO_2_-reducing methanogenesis. Therefore, *Methanothrix* likely plays an essential role in supporting multiple H_2_-independent electron disposal routes for organisms performing HS metabolism.

### Temperature-based differences

The coupling of HS catabolism with hydrolysis/HT catabolism, formate generation, oxygen respiration, and DIET was observed across all reactors, despite variation in temperature (Table S7). This indicates that the described phenomena may support HS metabolism at a wide temperature range. Across all studied temperatures, we observed Desulfobacterota and Spirochaetota FA degradation coupled with the expression of formate generation, oxygen respiration, and DIET (with the exception of Desulfobacterota at thermophilic temperature). Strict reliance on formate-generating FA degradation was only observed at mesophilic temperatures. Coupled expression of HS and HT AA degradation (with complementary formate/H_2_ generation) was also observed across all temperatures. However, some of these AA-degrading species were only observed to have high activity and express complete pathways at specific temperature ranges—UBP6, KSB1, Cloacimonadota, Fermentibacterota, Marinisomatota, Chloroflexota, Firmicutes_A, Firmicutes (~35 °C); WOR-3_A, Firmicutes_E (> 50 °C), Krumholtzibacterota (≤ 30 °C); Coprothermobacterota (> 40 °C); Myxococcota, Spirochaetota, Planctomycetota (< 50 °C), Caldisericota (~ 35 °C and > 50 °C). For methanogenesis, CO_2_-reducing methanogenesis by *Methanothrix* (potentially driven by DIET) was detected across all temperatures, but formate oxidation by *Methanothrix* and methyl reduction by Methanofastidiosa was only observed at temperatures below 50 °C. Thus, based on the available data, strategies for supporting HS metabolism and organisms that perform these challenging reactions differ between anaerobic digesters operated at different temperatures. However, analyses of more samples at non-standard temperatures (~35 °C) are necessary to better characterize temperature-based variation.

## Conclusion

**Fig. 5:**
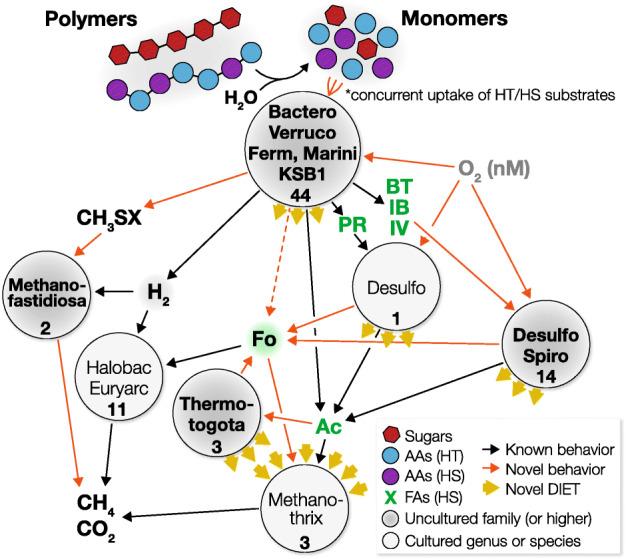
Updated scheme of methanogenic organic matter mineralization. Known and novel metabolic interactions and behaviors are shown (black and orange arrows, respectively). For each ecological niche, representative phyla and total number of species (number) associated with these phyla are shown. Ecological niches involving lineages uncultured at the family level or higher are indicated (bold with a gray background). Cascading degradation of polymers to monomers (sugars—red, H_2_ tolerant AAs—purple, H_2_ sensitive AAs—blue) generates metabolic intermediates whose degradation is H_2_-tolerant or H_2_-independent (black) and H_2_-sensitive (green letters). Novel electron transfer and syntrophic interactions involve formate as a key intermediate (green background) and DIET-mediated electric interactions (yellow arrows facing outward for electrogens and yellow arrows facing inward for electron-consuming species). Abbreviations: Bacteroidota (Bactero), Verrucomicrobiota (Verruco), Fermentibacterota (Ferm), Marinisomatota (Marini), Desulfobacterota (Desulfo), Spirochaetota (Spiro), Halobacterota (Halobac), Euryarchaeota (Euryarc), formate (Fo), acetate (Ac), propionate (PR), butyrate (BT), isobutyrate (IB), isovalerate (IV)

In methanogenic ecosystems, degradation of organic matter generates H_2_ as a central byproduct and necessitates microbial interactions between H_2_-generating organotrophic bacteria and H_2_-consuming methanogenic archaea. However, organotrophic metabolisms have diverse thermodynamic properties and many processes (i.e., HT catabolism) can generate H_2_ concentrations much beyond the thermodynamic limit of others (i.e., HS catabolism), which has not been addressed in previous models. Through metagenomic and metatranscriptomic analyses of multiple anaerobic digesters, we predict that uncultured organisms may employ unique strategies to drive thermodynamically competing metabolisms (Fig. 5)—parallel and broad-range HS and HT metabolism, a shift (often complete) from H_2_ to formate as a soluble electron carrier, respiration of low-concentration O_2_, DIET and formate exchange with *Methanothrix*, and interaction with high H_2_-affinity methanogenesis by *Ca.* Methanofastidiosum. The observed metabolic behaviors are likely tailored to the thermodynamic conditions in situ and quite distinct from cultured organisms. With such omics-based insights, future cultivation-based studies can be designed to verify and further characterize organisms that perform thermodynamically challenging catabolism under the in situ selective pressures (e.g., enrichment/cultivation of syntrophic degraders in the presence of both methanogens and H_2_-producing fermenters). The newly discovered metabolic strategies and ecology driving organic matter mineralization improve our understanding of carbon cycling in methanogenic ecosystems and foundational knowledge for innovation in biotechnology.

## Methods

### Sample collection and sequencing

Anaerobic digesters in 17 full-scale municipal wastewater treatment plants were selected for metagenomic and nine digesters were selected for metatranscriptomic sequencing to cover a wide range of operation temperature within the sequencing capacity (Table S2). As described previously [38], most digesters had an activated sludge process upstream while one analyzed reactor only had primary treatment upstream (USRA). Likewise, most digesters were operated at mesophilic temperatures (~35 °C), but JPHG and JPTR were operated at a slightly elevated temperature (~40 °C), JPHW and USRA at slightly lower temperatures (< 30 °C), and USOA and JPMR at thermophilic temperatures (> 50 °C). Several digesters (JPHW, JPNA, and USDV) were operated in series (same retention time) with the first digester treating primary/secondary clarifier sludge and the second treating sludge produced by the first. ADurb, JPHG, JPNA, USST, and USCA also treating other non-sewage-derived waste, including food waste and sludge from other sources. Waste treated by HKST had high salinity and sulfate content and were dosed with ferric chloride to suppress the release of sulfide (4000 to 6000 mg/L chloride concentration). HKYL also treated tannery industry wastewater containing high zinc and chromium concentrations. Sludge samples for DNA and RNA sequencing were collected simultaneously (same day and same reactor). Different sludge samples were taken at separate time points (e.g., 1 month apart), as documented previously [38]. Genomic DNA was extracted using the FastDNA SPIN Kit for Soil (MP Biomedicals, Carlsbad, CA, USA). RNA was extracted using acid-phenol/chloroform/isoamyl alcohol (125:24:1) and chloroform, precipitated by cold ethanol, and purified by DNase treatments [41]. DNA and RNA samples were dispensed in a barcoded plate and shipped on dry ice to the Joint Genomic Institute (JGI) in the Department of Energy for sequencing using the Illumina HiSeq-2500 1 TB platform and HiSeq-2000 1 TB platform for DNA and RNA sequencing respectively (2 × 151 bp). See the following Department of Energy Joint Genome Institute standard operating procedures for metagenomics and metatranscriptomics: Metagenome SOP 1064 and Metatranscriptome SOP 1066.1.

### Metagenomics and metatranscriptomics

Raw metagenomic paired-end reads were trimmed using BBDuk v38.08 (https://sourceforge.net/projects/bbmap/) (adapter trimming: ktrim=r, minlen=40, minlenfraction=0.6, mink=11, tbo, tpe, k=23, hdist=1, hdist2=1, ftm=5; filtering/trimming: maq=8, maxns=1, minlen=40, minlenfraction=0.6, k=27, hdist=1, trimq=12, qtrim=rl) and assembled with metaSPAdes v3.10 (-k 21,55,79,103,127) [64]. Metagenomic reads from the same bioreactors at different time points were assembled together. Reads were mapped to metagenomic sequences using BBMap of the BBTools package v38.26 (https://sourceforge.net/projects/bbmap/) using a 99% similarity cutoff (minid = 0.99) and otherwise default parameters. For metatranscriptomic sequences, trimming, read error correction, and read mapping were performed in the same way.

Metagenomic co-assemblies were binned into individual metagenome-assembled genomes (MAGs) using MetaBAT v0.26.3 (default parameters), MaxBin 2.1 (-min_contig_length 2500 -markerset 40), and MyCC (-t 2500 -lt 0.4) [65–67], and these binning results were further combined using Binning_refiner v1.2 [68]. Genome completeness and contamination were assessed using CheckM v1.0.1 (default parameters) [69], and taxonomy was estimated using GTDBtk v0.3.0 (GTDB release89; default parameters) [35]. MAGs acquired from different reactors were clustered into species based on a pairwise mutation distance cutoff of 0.05 calculated using Mash (dist -v 0.05 -l) [36]. Gene expression profiles for each species were calculated using representative genes selected by clustering genes of all associated MAGs using CD-HIT [70] (cutoff of 98% similarity). For gene expression analyses, BBMap-predicted RPKM (reads per kilobase of the transcript, per million mapped reads) values were normalized to the median non-zero expression level of all coding genes.

### Gene annotation and metabolic reconstruction

All genomes were annotated through a combination of Prokka v1.13 (kingdom = Bacteria or Archaea chosen based on phylogeny defined by GTDBtk) [71] and further manual curation. We specifically examined sugar degradation (18 types), amino acid degradation (20 types), electron transduction mechanisms (e.g., NADH:quinone oxidoreductase), respiration (O_2_ and nitrogen species), H_2_ metabolism, formate metabolism, and polymer hydrolysis (glycosylhydrolase, extracellular peptidase, and extracellular lipase families). The curation involved functional domain analysis through CD-Search with its corresponding conserved domain database [72, 73]; signal peptide and transmembrane domain prediction through SignalP v4.1 (default parameters) [74]; carbohydrate-active enzyme, peptidase, and lipase prediction through dbCAN 5.0 [75], MEROPS [76], and lipase engineering database [77]; and hydrogenase annotation with assistance from HydDB [78] with default parameters. In addition, to further verify the function, we compared the sequence similarity of each gene to a database containing enzymes with experimentally verified catalytic activity and genes with extensive genetic, phylogenetic, and/or genomic characterizations with a 40% amino acid similarity cutoff. For enzymes that have divergent functions even with a 40% similarity cutoff (e.g., [FeFe] and [NiFe] hydrogenases, 2-oxoacid oxidoreductases, glutamate dehydrogenases, and sugar kinases), phylogenetic trees were constructed with reference sequences to identify the association of the query sequences to phylogenetic clusters containing enzymes with characterized catalytic activity. For hydrogenases (e.g., FeFe [HydABC, HndABCD] and bidirectional [Ech, Mbh, Hyb, Hox, Hup, sulfhydrogenase] NiFe hydrogenases), formate dehydrogenases (e.g., Fdh-H [FdhA, FdhAB, FdhA-HydBC] and Fdh-N [Fdn/oGHI] types), and electron transduction complexes (e.g., Rnf and Nqr) that are composed of multiple subunits and tend to co-localize in the genome, we only annotated the function of the complex if all subunits were identified in an operon or the operon appeared to be divided onto two contigs (i.e., two ends of an operon on the ends of two contigs). Pili were annotated to be conductive for pilA genes containing many aromatic residues (≥ 9% of total peptide length) relatively evenly distributed across the length of the protein (every 20 amino acids) as described in a previous study [79]. Membrane-bound or extracellular multi-heme cytochromes were annotated for proteins encoding transmembrane or N-terminal signal peptides respectively and multiple heme-binding sites.

In addition to gene annotation, metabolic capacities and traits (e.g., sugar and AA catabolism) were predicted based on the strict criteria that all enzymes necessary for the pathway could be identified. It is critical to be cautious in annotating anaerobic metabolism due to (i) the difficulty in the annotation of enzymes and pathways in specialized anaerobic metabolisms and (ii) the ambiguous directionality of catabolic enzymes and pathways. For example, genes and pathways for propionate catabolism are nearly indistinguishable from those for propionate fermentation. Similarly, many amino acid degradation genes and pathways can also be used for biosynthesis. Thus, an anaerobic catabolic pathway was included in the analysis when the target genome harbored a complete pathway for substrate oxidation and electron transfer reactions compatible with the re-oxidation of all electron carriers involved. For example, if an organism encodes oxidation of an AA that produces one NADH and one NADPH per substrate and has a ferredoxin-dependent hydrogenase, the AA catabolism is only predicted if the organism also encodes oxidoreductases/dehydrogenases that can transfer electrons from both NADH and NADPH to ferredoxin. To further confirm the directionality, we determine whether the predicted pathway (i) can also recover energy (e.g., generate ATP or proton motive force) and (ii) is catabolism-specific in biochemically characterized isolates or involves enzymes that are known to be used in the catabolic direction for steps (see Table S1). For some pathways, the directionality cannot be determined by sequence data alone (noted in Table S1 and Fig. 3). Based on the metabolic capacities predicted as above, we also define the total metabolic capacity for each species. Organisms from the same species can have different metabolic capacities, so metabolic capacities were predicted for each MAG prior to clustering into species to avoid creating “chimeric” metabolic reconstructions.

Metatranscriptomic-based activity of each metabolic pathway was predicted with strict criteria—expression of all genes involved in the pathway at a normalized expression level (RPKM of target gene divided by the median RPKM of all genes belonging to the target MAG) ≥ 1 by the target species-level MAG cluster in a single reactor averaged over the triplicate metatranscriptomes. Although a species-level cluster of MAGs can contain more metabolic capacities than the individual MAGs, the false prediction is not anticipated as only genes that are present would be detected and reads were mapped with high stringency (99% similarity).

To identify potential correlations between metabolic capacities, principal correspondence analysis was performed using R and the R packages FactoMineR and ggplot2 [80–82]. For this, a matrix containing the species-level MAG clusters with their corresponding the presence (value of 1), absence (value of 0), or diversity (see the following sentence) of each metabolic capacity was constructed. For diversity, the number of protein families (proteases and glycosyl hydrolases) or the number of pathways (fatty acid, AA, and sugar degradation) present in the target MAG cluster were used as values. Confidence ellipses (95%) were also plotted using the ggplot2 package (stat_ellipse). Similarly, to further identify relationships between metabolic activities, principle correspondence analysis was conducted for a matrix containing the species-level MAG clusters with expression levels of representative genes from individual pathways or diversity of pathways expressed for a particular category of metabolism. The following values were employed—for proteases and glycosylhydrolases, the number of protein families expressed in at least one reactor; for fatty acid, AA, and sugar degradation, the number of pathways expressed in at least one reactor; for electron transfer/energy conservation pathways (Rnf, Nfn, Fix, Efd, and FloxHdr), the number of pathways expressed in at least one reactor; for H_2_ and formate generation, the highest normalized expression level (RPKM normalized to species’ non-zero median expression level) detected for hydrogenase and formate dehydrogenase catalytic subunits across all reactors; and, for O_2_ respiration, the highest normalized expression level of any cytochrome bd oxidase subunit across all reactors. Although the principal correspondence analysis for the metatranscriptome-based metabolic activity was based on values spanning across all analyzed reactors, the observed and discussed correlations were further verified based on activities in individual reactors (see Table S7 and S16). Pearson correlation and Student’s *t* test calculations were performed using Microsoft Excel functions Pearson() and T.DIST().

## Supplementary information

**Additional file 1: Supplementary figure S1**. H_2_-dependent Gibbs free energy yield of representative metabolisms. ∆G values (∆G_reaction_ + ∆G_ATPyield_; vertical axis) with varying H_2_ concentrations (horizontal axis) are shown for FA degradation (green), HS AA degradation (purple), select HT AA degradation (blue), glucose degradation (red), methylated thiol-dependent methanogenesis (black), and CO_2_-dependent methanogenesis (black). Maximum H_2_ concentrations are shown for FA and HS AA degradation pathways (colored arrows on horizontal axis). See Figure 3 for conditions used for calculating ∆G.

**Additional file 2: Supplementary figure S2**. GTDBtk-based phylogenomic tree of (left) bacterial and (right) archaeal MAGs. Phyla for which MAGs were recovered are marked gray. The number of species (*i.e.*, MAG clusters) associated with each phylum and sub-lineage are shown

**Additional file 3: Supplementary figure S3**. Activity of microbial species in anaerobic digesters. For each species, the ratio of metatranscriptome (MetaT)-based activity (percentage of reads mapped to specific species out of all reads mapped to active species) and metagenome-based abundance in the reactor where the species displays the highest MetaT-based activity was calculated. (left) For each phylum, the minimum, 1^st^ quartile, median, 3^rd^ quartile, and median + 1.5 x interquartile range are shown as a box-whisker plot. Outliers are not shown. The minimum value is indicated for phyla whose minimum is outside of the plot’s range. (right) The number of active (blue) and less active (yellow) species (≥0.4% mapped transcriptome for bacteria and ≥0.3% for archaea) are shown for each phylum. The values are indicated for phyla whose values exceed the plot’s range.

**Additional file 4: Supplementary figure S4**. Example of metabolic reconstruction for candidate phylum KSB1 species cluster 1285 (representative MAG USDE125). (a) Overall electron flow and energetics of amino acid metabolism, (b) the electron transfer complexes, hydrogenases, and formate dehydrogenases surveyed (identified complexes marked red), and (c) metabolic pathways surveyed (complete pathways expressed marked red; catabolic pathways and catabolic/irreversible enzymes marked with red diamonds) are shown

**Additional file 5: Supplementary figure S5**. Comparison of predicted metabolic potentials of individual MAGs and MAG species clusters they belong to**.** For each species cluster and each metabolic category (left to right – amino acid catabolism, fatty acid catabolism, and extracellular hydrolytic enzymes [glycosylhydrolases, peptidases, and lipases]), the maximum observed number of metabolic pathways among MAGs belonging to a single species cluster was divided by the total observed pathways across all MAGs belonging to a single species cluster.

**Additional file 6: Supplementary Tables**.

## Data Availability

All metagenome-assembled genomes (MAGs) are available in the GenBank under BioProject PRJNA321808 (deposited annotations are non-curated automatic predictions by Prokka). All raw metagenome and metatrancriptome data and assemblies are available on the Joint Genome Institute Integrated microbial genome and metagenome (IMG/M) database (see Table S2 for project IDs). The raw data is available in the Joint Genome Institute Genome Portal (https://genome.jgi.doe.gov/portal/).

## Abbreviations

AA: Amino acid
FA: Fatty acid
HT: H_2_-tolerant
HS: H_2_-sensitive
MAG: Metagenome-assembled genome
ECM50: A species expressing the **c**omplete **m**etabolic pathway(s) in at least 50% of the studied reactors where it comprised ≥ 0.05% of the mapped metatranscriptome
RPKM: Reads per kilobase of transcript per million mapped reads
DIET: Direct interspecies electron transfer
